# Hidden in plain sight: How helminths manage to thrive in host blood

**DOI:** 10.3389/fpara.2023.1128299

**Published:** 2023-03-10

**Authors:** Maude Dagenais, Lucienne Tritten

**Affiliations:** ^1^ Institute of Parasitology, McGill University, Ste-Anne-de-Bellevue, QC, Canada; ^2^ Medical Parasitology and Infection Biology, Swiss Tropical and Public Health Institute, Allschwil, Switzerland; ^3^ University of Basel, Basel, Switzerland; ^4^ Institute of Parasitology, Vetsuisse Faculty, University of Zurich, Zurich, Switzerland

**Keywords:** helminth, blood-borne, complement system, coagulation, immune evasion, innate immunity, host factors

## Abstract

Parasitic helminths have evolved a plethora of elegant stratagems to regulate and evade the host immune system, contributing to their considerable persistence and longevity in their vertebrate hosts. Various mechanisms to achieve this state have been described, ranging from interfering with or actively modulating host immune responses to hiding from immune recognition. Because they damage surrounding vessels and disturb blood flow, blood-borne and blood-feeding parasites in particular must deal with much more than immune effector cells. Management of the host complement system and coagulation cascade, as well as the development of processes of hiding and masking, represent hallmarks of life in blood. Here we review recent findings on putative evasion strategies employed by blood-borne parasitic helminths, focusing on the interaction with and utilisation of host serum components by nematodes and trematodes.

## 1 Introduction

Parasitic helminths remain extremely prevalent, infecting over 1.15 billion people based on estimates from 2017 (James et al., 2018), causing a variety of illnesses. One particularity of these parasites is their capacity to establish chronic infections, persisting in mammalian hosts for many months to years or even decades. This is due to their remarkable ability to evade and disrupt the broad variety of immune defence mechanisms deployed against them (Maizels and McSorley, 2016), which not only results in prolonged infections, but also greatly complicates control efforts, including the development of effective vaccines. Immunomodulatory strategies utilised by parasitic helminths have been reviewed extensively (Reviewed in McSorley et al., 2013; Maizels et al., 2018; Zakeri et al., 2018; Wiedemann and Voehringer, 2020, and Acharya et al., 2021). The circulatory system is home to multiple immune effectors and enables transport of leukocytes between tissues and lymph nodes, thus serving as a pipeline for the immune system (Chaussabel et al., 2010). Consequently, blood-borne pathogens are particularly vulnerable to immune recognition and attack and must cope with or modulate the host immune system to establish a chronic infection. This is particularly true for extracellular pathogens such as helminths, large multicellular organisms that cannot hide inside host cells, and therefore are predicted to disturb blood flow (Bloch, 1980; Mebius et al., 2013), damage vessels through feeding and body migration, and interfere with primary and secondary haemostasis, fibrinolysis, and vascular tone (Mebius et al., 2013). Despite this seemingly highly disruptive lifestyle, blood-borne helminths surprisingly elicit very little inflammation and generally dodge immunological attacks targeted at them in permissive hosts (Maizels and McSorley, 2016). A number of immune evasion mechanisms have been described for blood-borne parasitic helminths (Pearce and Sher, 1987; Maizels et al., 1993; Wiedemann and Voehringer, 2020). The hemostatic system comprises both procoagulant and anticoagulant functions that prevent blood loss at sites of injury, and importantly play critical roles in innate immunity. Here we review recent findings on the modulation by blood-borne helminths of less well-known host functions at the interface between immunity and haemostasis, as well as appropriation of host molecules to facilitate evasion of host immunity.

### 1.1 Co-opting of host molecules by blood-borne helminths

The innate immune system has evolved strategies to discriminate between healthy and abnormal self cells and non-self cells, which rely on recognition of markers (self, non-self, and pathogen-associated) by receptors. Binding of self-markers suppresses an immune response, whereas non-self markers or pathogen-associated molecular patterns (PAMPs) elicit a reaction (Medzhitov and Janeway, 2002). Many pathogens have been proposed to exploit these strategies by disguising with host self markers and/or by masking their PAMPs, preventing activation of signalling pathways that induce antipathogen effector responses and inflammation (Joiner et al., 1986; Pereira-Chioccola et al., 2000; Vogl et al., 2008; Brouwer et al., 2022). Early work by Smithers et al. (1969) demonstrated the presence of host antigens on the surface of adult schistosomes; many other researchers have since reported the acquisition of host molecules by this parasite at various stages of development. Upon invasion of the mammalian host, newly transformed schistosomula shed their glycocalyx, which is replaced by a heptalaminate membrane tegument. This new tegument allows selective adhesion of host factors *via* surface receptors. Notable examples include binding of IgG to Fc receptors located on the tegument (Kemp et al., 1977; Torpier et al., 1979; Yong and Das, 1983; Loukas et al., 2001), hypothesised to be preventing interaction of IgG with Fc receptors on immune effector cells; adsorption of low-density lipoproteins, possibly restricting binding sites available for antibodies (Rumjanek et al., 1988; Chiang and Caulfield, 1989a; Chiang and Caulfield, 1989b), and C3 decay-accelerating factor, possibly conferring protection against complement-mediated killing (Horta et al., 1991). Importantly, though functions have been proposed for the co-opting of such host molecules by the parasites, they remain speculative. Other host-derived factors adhere to the schistosome tegument, including but not limited to complement components (Santoro et al., 1980; Tarleton and Kemp, 1981; Skelly and Wilson, 2006), though their exact function is not known. Rare examples in nematodes include acquisition of host complement regulators factor H (Steenbeek et al., 2018) and C4b-binding proteins (C4BP) by microfilariae of *Loa loa* (Haapasalo et al., 2009) and fH by *Onchocerca volvulus* microfilariae (Meri et al., 2002), which transit through host blood. Microfilariae-bound fH and C4BP are functionally active and inhibit complement activation, protecting the worm from subsequent attacks from phagocytes relying on complement components as receptors (Meri et al., 2002; Haapasalo et al., 2009). However, perhaps owing to the vastly different properties of the tegument compared to nematode cuticles (a syncytium bounded externally by two lipid bilayer membranes permitting nutrient transport *vs* a thick acellular barrier that is not actively absorptive (Pappas, 1988; Skelly et al., 1998; Skelly et al., 2014), instances of utilisation of host-derived molecules to avoid immune recognition have largely been described in the context of infection with trematodes, with schistosomes being the most studied. Most of these findings are several years old and have been reviewed recently (Hambrook and Hanington, 2021); however, recent data suggest that schistosomes co-opt yet more host molecules to coat their extracellular vesicles (EVs) (Dagenais et al., 2022).

Sialic acid (SA) residues were discovered to be associated with adult *Schistosoma mansoni* EVs (Dagenais et al., 2021). SA are acidic nine-carbon monosaccharides frequently present as terminal residues of glycoconjugates in higher animals. They regulate many biological processes, such as cell-cell and cell-extracellular matrix interactions, either by mediating interaction or by masking recognition sites, and are believed to be a self marker (Schauer, 1985; Schauer and Kamerling, 2018). Helminths do not appear to possess SA synthesis enzymes (Hokke and van Diepen, 2017; McVeigh et al., 2018; Schauer and Kamerling, 2018). Several pathogens utilise SA as part of their invasion strategy, including viruses (Rempel et al., 2008), bacteria (Corfield, 1992; Shakhnovich et al., 2002; Bouchet et al., 2003; Sohanpal et al., 2004; Sohanpal et al., 2007; Mehr and Withers, 2016), fungi (Alviano et al., 1999; Eneva et al., 2021), and protozoan parasites, such as *Trypanosoma cruzi* (Giorgi and De Lederkremer, 2011)*, T. brucei* (Schauer and Kamerling, 2011), and *Leishmania* spp. (Previato et al., 1985), obtaining them either by *de novo* synthesis or by incorporating pre-formed SA residues from the host (Severi et al., 2007; Cavalcante et al., 2021). Organisms unable to synthesise SAs depend on sialidases (also known as neuraminidases), which cleave terminal SA from host sialoglycans, or trans-sialidases, which hydrolyse SAs and direct the transfer of host sialyl residues onto their own asialoglycoconjugates (non-sialylated glycoconjugates) (Corfield, 1992; Schauer and Kamerling, 2011; Mehr and Withers, 2016). Many advantages have been attributed to the coating of pathogens with SA residues, including concealing surface antigens and adhesion to and invasion of host cells (Schenkman et al., 1991; Rubin-de-Celis et al., 2006; Campetella et al., 2020). Sporadic reports suggest the presence of SA in helminths, which have generally been attributed to contamination with host molecules (Warren, 1963; Khoo et al., 1997; Lee et al., 2005; Ravidà et al., 2016). However, the detection of SA on *S. mansoni* EVs after an elaborate density-based EV isolation process suggests that SA is associated with EVs rather than simply present in the sample (Dagenais et al., 2021). Mass spectrometry analyses of EV sialoglycoproteins revealed the presence of mammalian serum glycoproteins (Dagenais et al., 2022). It is thus conceivable that schistosomes, and perhaps other helminths, exploit host sialoglycoconjugates, in the form of whole glycoproteins, to aid in the infection process. Coating of parasite EVs with host sialylated proteins could benefit the parasite in many ways, including masking non-self antigens and coating with self molecules to increase their half-life in the host, allowing them to reach more distant cellular targets *in vivo*. SA-bearing EVs could also dampen immune responses and inhibit immune cells *via* interactions with host surface molecules such as SA-binding immunoglobulin (Ig)-like lectins (Siglecs), which are expressed on most leukocytes, resulting in inhibition of cell activation (Crocker et al., 2007).

**Figure 1:**
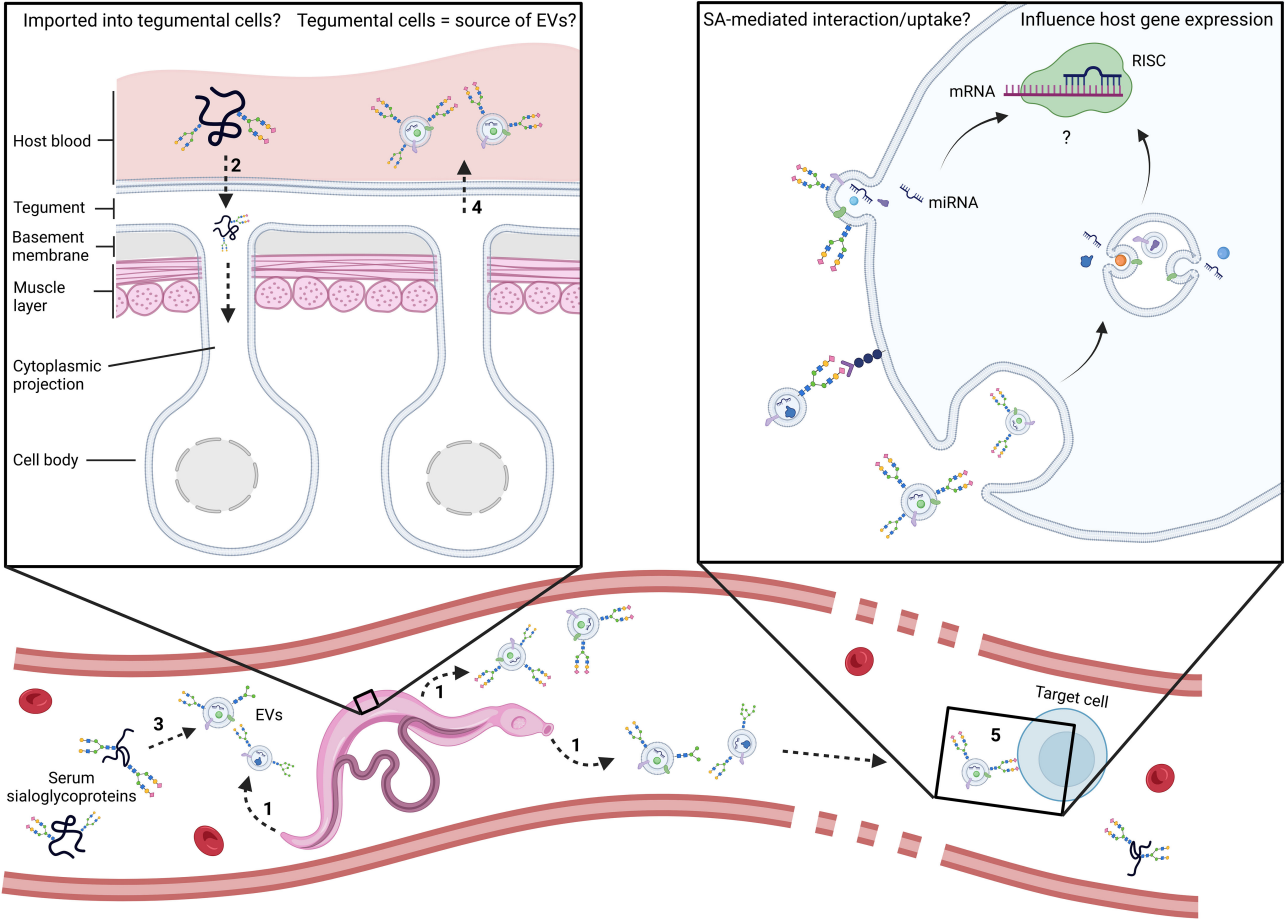
Schematic representation of the main outstanding questions related to the utilisation of host serum sialoglycoproteins by adult schistosomes. Are EVs coated passively or as an active and specific process? At which point do host glycoconjugates become associated with parasite EVs? In the trematodes *Schistosoma mansoni* and *Fasciola hepatica*, evidence points to the tegument, digestive and excretory systems as sources of EVs (1) (de la Torre-Escudero et al., 2016; Bennett et al., 2020; Dagenais et al., 2021; Bennett et al., 2022). In addition, lectin histochemistry of whole adult worms with SNA-I, a lectin which recognises structures with α2-6-linked terminal SA, strongly labelled sub-tegumental cell bodies of the parasite (Dagenais et al., 2021), suggesting uptake of SA into those cells. Consequently, it is plausible that schistosomes might acquire serum glycoproteins *via* the tegument. Could schistosomes have receptors on sub-tegumental cell surfaces that recognize sialic acid as a signal for endocytosis (2)? Alternatively, the coating of EVs might occur upon their release in host blood (3). Do EVs emanate from sub-tegumental cells (4)? Does SA mediate interaction with target cells and/or EV uptake (5)? Future research should address these outstanding questions and investigate the involvement of SA in cellular interaction and EV uptake. miRNA, microRNA; mRNA, messenger RNA; RISC, RNA-induced silencing complex.

It is increasingly recognised that the glycosylation profile of EVs plays a crucial role in their biodistribution and uptake and thus ultimately affects their functions (Williams et al., 2018; de la Torre-Escudero et al., 2019; Williams et al., 2019; Kuipers et al., 2020). The glycobiome of mammalian-sourced EVs is generally enriched in sialylated glycoconjugates (Williams et al., 2018) and their removal leads to altered biodistribution *in vivo* (Royo et al., 2019), suggesting the importance of SA in EV migration and cellular interaction. It is interesting to consider that blood flukes might hijack this strategy to ensure optimal distribution of their EVs in the host, mediate specific interactions with host cells, and facilitate internalisation of EVs by target cells where they can exert their functions, such as by miRNA-driven translational repression of host genes (Buck et al., 2014; Meningher et al., 2020). Some outstanding questions are presented in **Figure 1**.

### 1.2 Interference with host hemostasis

**Figure 2:**
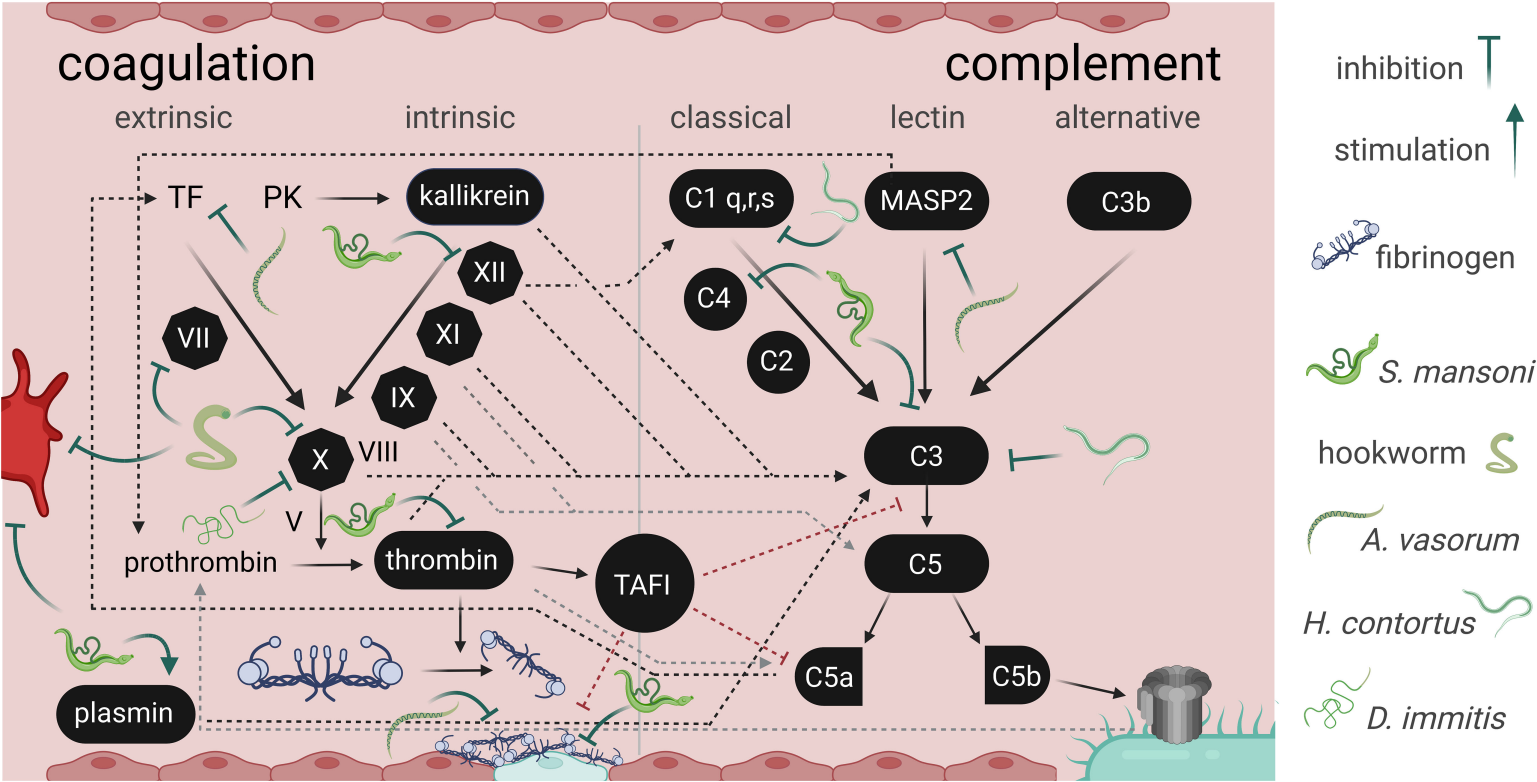
Interference between parasitic helminths with the secondary hemostasis and complement cascade. The image depicts some (non-exhaustive) points of interactions between helminth E/S products and the interconnected coagulation cascade and complement system, an integral part of innate immunity. While parasite molecules have inhibitory effects on host processes, *Schistosoma mansoni* stimulates plasmin activation to promote fibrinolysis (Mebius et al., 2013). The trematode also indirectly inhibits fibrin clot formation and stabilisation, interacts with FXII, thrombin, and complement C3 and C4 (Mebius et al., 2013; Da’dara et al., 2017; Wang et al., 2017; Wang et al., 2018). Hookworms interact with FVII and FX (reviewed in Abuzeid et al., 2020). *Haemonchus contortus* impairs C1q and C3 functions (Vedamurthy et al., 2015). *Dirofilaria immitis* was shown to decrease FX activity (Diosdado et al., 2020). *Angiostrongylus vasorum* interferes with fibrin clot stabilization, with tissue factor, and mannose-binding lectin associated protease 2 (MASP2) (Gillis-Germitsch et al., 2021; Tritten et al., 2021b). Interference with platelet activation or aggregation (bright red cell; primary hemostasis) was described for hookworm and *S. mansoni* products (Del Valle et al., 2003; Elzoheiry et al., 2018a). The coagulation and complement cascades are depicted according to Danckwardt et al. (2013). TF, tissue factor; PK, prekallikrein; TAFI, thrombin activatable fibrinolysis inhibito; MASP2, Mannose-binding lectin-associated serine protease 2.

The three events that may lead to activation of coagulation according to the Virchow triad are: i) hypercoagulable state of blood plasma, ii) disruption/alteration of normal blood flow (stasis), and iii) damage/impairment of the endothelium (Kushner et al., 2019). As large pathogens occupying a significant portion of a small vessels lumen, blood-dwelling helminths may initiate coagulation *via* each of these three events. Therefore, besides adaptations to evade the host immune system, parasites have evolved strategies to prevent blood coagulation, which would compromise their ability to feed and move freely, hampering their overall survival (**Figure 2**). How *S. mansoni* interferes with the host hemostatic system was reviewed previously (Mebius et al., 2013). Several schistosome molecules contained in the tegument are predicted to have enzymatic activity, which could lead to platelet activation inhibition, e.g., *via* degradation of extracellular adenosine diphosphate (ADP; Da’dara et al., 2014; Skelly et al., 2022) or triggering release of platelet activation inhibitors such as prostacyclin. Similarly, mechanisms behind fibrin generation and formation of a stable blood clot have been shown or proposed to be inhibited and regulated through the production of anticoagulants (e.g., Sm22.6, heparin-like molecules, etc.) (Mebius et al., 2013). Activating fibrinolysis represents another way for schistosomes to prevent entrapment by the host. Several endogenous factors and schistosome-derived proteins stimulate proteolytic activation of plasminogen into the fibrin-degrading enzyme plasmin. At least ten *S. mansoni* proteins bind plasminogen. Among those, enolase, glyceraldehyde 3-phosphate dehydrogenase (GAPDH), and a serine protease (SmSP2) facilitate the conversion of plasminogen to plasmin (via tissue plasminogen activator), initiating fibrinolysis (Yang et al., 2010; Mebius et al., 2013; Figueiredo et al., 2015; Leontovyč et al., 2018). The same was observed for excretory/secretory (E/S) proteins released by the blood-borne nematodes *Dirofilaria immitis* and *O. volvulus* (Jolodar et al., 2003; González-Miguel et al., 2012). Two functional calpains expressed on the schistosome tegument can cleave host fibronectin (a stabiliser of fibrin clots) and high molecular weight kininogen (a cofactor involved in the conversion of FXII in the intrinsic coagulation pathway) (Wang et al., 2017; Wang et al., 2018). Similarly, an alkaline phosphatase (SmAP) on the worm’s surface degrades sphingosine-1-phosphate (S1P), a molecule with pro-inflammatory and pro-coagulant functions, among others (Elzoheiry et al., 2018b). Hookworms (*Ancylostoma* spp. and *Necator americanus*) feed on blood; despite not being blood-borne parasites, they must prevent clot formation at the host intestinal attachment site and during the passage of blood through their gut. Hookworms secrete powerful inhibitors of platelet aggregation, which hinder platelet adherence to fibrinogen, and several anticoagulants (interacting with e.g., FVIIa and FXa) with distinct modes of action affecting the coagulation cascade (Abuzeid et al., 2020). The dog heartworm *D. immitis* can survive in its host circulatory system for years (Newton, 1968). Like many parasitic helminths, it secretes proteins in the serine protease inhibitor (serpin) family, capable of immune regulation and inhibition of the coagulation cascade (Knox, 2007; Molehin et al., 2012). *Dirofilaria immitis* E/S proteins partially alter the host coagulation cascade and reduce the activity of FXa (Diosdado et al., 2020), which plays a pivotal role at the intersection between the intrinsic and extrinsic pathways of coagulation and stimulates conversion of fibrinogen into fibrin (Adams and Bird, 2009). One *D. immitis* protein in particular, homologous to *Brugia malayi* serpin 6, was proposed to form an inhibitory complex with FXa (Diosdado et al., 2020).

Alterations on the complement and coagulation cascades could harm the host. Canine infections with the lung and heart parasite *Angiostrongylus vasorum* are associated with coagulopathies with frequently fatal outcomes (Glaus et al., 2016). Infection decreases levels of some complement lectin pathway components (mannose-binding lectin (MBL)-associated serine protease (MASPs) and ficolin 1) as well as coagulation factors such as Factor XIII subunit B (Tritten et al., 2021b), which stabilises fibrin clots and accelerates fibrinogen cross-linking (Souri et al., 2015). Paralleling this, inhibition of MASPs and complement activation by *Fasciola hepatica* serpins was described (De Marco Verissimo et al., 2022). Additionally, proteomic analysis revealed that E/S proteins released by this parasite contain several putative modulators of host coagulation (Gillis-Germitsch et al., 2021). Similarly, humans infected with *S. mansoni* showed lower platelet counts and significantly more coagulation abnormalities than uninfected patients (Eyayu et al., 2020). In line with this, mouse blood exposed to adult *S. mansoni* worms *in vitro* clotted more slowly and yielded relatively poor, though stable, thrombi (Da’dara et al., 2016).

### 1.3 Modulation of complement: Meddling in both coagulation and immunity

The coagulation cascade and the complement system are interconnected (**Figure 2**), along with other important immune mechanisms beyond the scope of this review, such as NETosis (formation of neutrophil extracellular traps) (de Bont et al., 2019). Some coagulation factors, such as FXII, activate the complement cascade (Tsang et al., 1977). Interference with host FXII activity by schistosomes may therefore be doubly beneficial for the pathogen: to prevent clot formation and to evade the innate immune system (Mebius et al., 2013). Schistosomes engage complement in human serum and rapidly degrade selected complement proteins such as C3, C4 and factor B, which may help explain the worm’s refractoriness toward complement-mediated attack (Da’dara et al., 2017). Indeed, helminths residing in host blood have evolved a variety of strategies to evade the complement system. These range from recruitment of host regulatory proteins to molecular mimicry or release of inhibitors of complement activation (reviewed in Shao et al., 2019). The latter category in particular is well known in helminths: *B. malayi* and *N. americanus*, for instance, release calreticulin, which inhibits the lectin pathway by binding host mannose-binding lectin (Brown et al., 1999). GAPDH secreted by *Haemonchus contortus*, another blood-feeder, binds to C1q and C3, shutting down complement activation (Vedamurthy et al., 2015). The complement binding capacities of *D. immitis* E/S proteins shed by microfilariae are inhibited by macrocyclic lactone anthelmintics (Staniunas and Hammerberg, 1982). The relationship between immunomodulatory properties of E/S proteins and the anthelmintic effects of macrocyclic lactones was established with evidence suggesting that these drugs act by suppressing the parasite’s ability to secrete molecules that enable evasion of the host immune system, likely including the complement system (Moreno et al., 2010).

## 2 Discussion

Cross-talk at the host-parasite interface is dynamic. The composition of E/S material varies both qualitatively and quantitatively depending on parasite developmental stage, and likely based on the host species and tissue it occupies (Tritten et al., 2021a; Castro-Borges and Wilson, 2022). Adding another layer of complexity, the composition of EVs may change following their release in the host. Indeed, the protein corona at the surface of EVs may be formed during EV biogenesis but may also be acquired in the extracellular space, such as host blood, contributing to their functional identity (Buzas, 2022). Helminth EVs may indeed acquire some elements from the host to decorate their EV surface, such as serum sialoglycoproteins (Dagenais et al., 2022), which may mediate important functional activities as well as confound our ability to differentiate factors that contribute to host modulation from those that do not. The intricacies of helminths’ ability to hide in plain sight remain poorly understood, particularly when it comes to their interaction with host blood components, as most research focuses on interaction with immune cells.

As illustrated by their divergent life histories, helminth pathogens are complex and present an impressive genomic diversity (International Helminth Genomes Consortium, 2019). Despite significant advances in genomics, our grasp of the biology of these parasites and what allows them to survive in the bloodstream of other organisms is limited, and the development of effective treatments is still impacted by methodological limitations. While infectious disease research has greatly benefited from transgenesis in the past decade (e.g., for vaccine design), genome manipulation in parasitic helminths still lags behind that of many other pathogens profiting from state-of-the-art advances (Quinzo et al., 2022).

Currently, functional studies of molecules defining the nature of parasitism rely on extensive ‘omics’ analyses. Among molecules released at the host-parasite interface, the soluble protein fraction is best characterised (Tritten et al., 2021a). Recent technological advances have enabled the study of other helminth E/S components, providing insights into the importance of miRNAs, lipids and glycans in the host-parasite crosstalk, among others. Integrating information from genomics, transcriptomics, immunomics, and new disciplines such as proteogenomics, may illuminate fundamental biology and lead to translation into therapeutic applications (Sotillo et al., 2017).

Access to high-throughput approaches will benefit the study of helminth-host interactions. For instance, targeted proteomics experiments allow the precise detection of enzymatic cleavage in a protein sequence, as in N-terminome analyses (Kleifeld et al., 2010). Such assays will be useful to study host-parasite interactions that degrade host material such as coagulation factors or complement components, offering both depth and high-throughput capabilities. Detailed investigations of other specialised proteomes may highlight potential drug targets. Analysis of the *S. mansoni* phosphoproteome revealed important features of female worm biology (Hirst et al., 2020). Phosphorylation is an essential post-translational modification for signal transduction within cells, and is instrumental to many cellular processes. Protein kinases and phosphatases catalyse the addition and removal of phosphate groups, respectively, thereby altering the properties of target proteins. (Ubersax and Ferrell, 2007). Protein kinases represent interesting drug targets, and several inhibitors have been approved for human use in other contexts (Santos et al., 2017); whether these could facilitate the design of drugs against schistosomes remains to be investigated (Wu et al., 2021).

To conclude, recent technological developments open new opportunities to better understand survival strategies employed by helminths intimately exposed to host blood and defences. In the past few years, few research efforts have been dedicated to the interactions between parasites and host blood components. However, it is expected that parasite E/S proteins have not yet revealed all their ‘moonlighting’ potential as determinants of parasitism (Tritten et al., 2021a) and the extensive overlap between host hemostasis and immune pathways merits renewed attention.

## Author contributions

Conceptualisation, MD, LT. Writing—original draft preparation, MD, LT. Writing—review and editing, MD, LT. All authors contributed to the article and approved the submitted version.

## References

[B1] Abuzeid A. M. Zhou X. Huang Y. Li G. (2020). Twenty-five-year research progress in hookworm excretory/secretory products. Parasites Vectors 13, 1–18. doi: 10.1186/s13071-020-04010-8 32171305 PMC7071665

[B2] Acharya S. Da’dara A. A. Skelly P. J. (2021). Schistosome immunomodulators. PloS Pathog. 17, e1010064. doi: 10.1371/journal.ppat.1010064 34969052 PMC8718004

[B3] Adams R. L. Bird R. J. (2009). Coagulation cascade and therapeutics update: Relevance to nephrology. part 1: Overview of coagulation, thrombophilias and history of anticoagulants. Nephrology 14, 462–470. doi: 10.1111/j.1440-1797.2009.01128.x 19674315

[B4] Alviano C. S. Travassos L. R. Schauer R. (1999). Sialic acids in fungi. Glycoconjugate J. 16, 545–554. doi: 10.1023/A:1007078106280 10815991

[B5] Bennett A. P. de la Torre-Escudero E. Dermott S. S. Threadgold L. T. Hanna R. E. Robinson M. W. (2022). *Fasciola hepatica* gastrodermal cells selectively release extracellular vesicles *via* a novel atypical secretory mechanism. Int. J. Mol. Sci. 23, 5525. doi: 10.3390/ijms23105525 35628335 PMC9143473

[B6] Bennett A. P. de la Torre-Escudero E. Oliver N. A. Huson K. M. Robinson M. W. (2020). The cellular and molecular origins of extracellular vesicles released by the helminth pathogen, *Fasciola hepatica* . Int. J. Parasitol. 50, 671–683. doi: 10.1016/j.ijpara.2020.03.015 32569641

[B7] Bloch E. H. (1980). *In vivo* microscopy of schistosomiasis. II. migration of *Schistosoma mansoni* in the lungs, liver, and intestine. Am. J. Trop. Med. Hygiene 29, 62–70. doi: 10.4269/ajtmh.1980.29.62 7352629

[B8] Bouchet V. Hood D. W. Li J. Brisson J.-R. Randle G. A. Martin A.. (2003). Host-derived sialic acid is incorporated into *Haemophilus influenzae* lipopolysaccharide and is a major virulence factor in experimental otitis media. Proc. Natl. Acad. Sci. 100, 8898–8903. doi: 10.1073/pnas.1432026100 12855765 PMC166410

[B9] Brouwer S. Jespersen M. G. Ong C.-L. Y. De Oliveira D. M. Keller B. Cork A. J.. (2022). *Streptococcus pyogenes* hijacks host glutathione for growth and innate immune evasion. Mbio 13, e00676–e00622. doi: 10.1128/mbio.00676-22 35467425 PMC9239160

[B10] Brown A. Kasper G. Mcelroy P. Loukas A. Hewitt C. Berry C.. (1999). A hookworm allergen which strongly resembles calreticulin. Parasite Immunol. 21, 439–450. doi: 10.1046/j.1365-3024.1999.00238.x 10476053

[B11] Buck A. H. Coakley G. Simbari F. Mcsorley H. J. Quintana J. F. Le Bihan T.. (2014). Exosomes secreted by nematode parasites transfer small RNAs to mammalian cells and modulate innate immunity. Nat. Commun. 5, 1–12. doi: 10.1038/ncomms6488 PMC4263141 25421927

[B12] Buzas E. I. (2022). Opportunities and challenges in studying the extracellular vesicle corona. Nat. Cell Biol. 24, 1322–1325. doi: 10.1038/s41556-022-00983-z 36042293

[B13] Campetella O. Buscaglia C. A. Mucci J. Leguizamón M. S. (2020). Parasite-host glycan interactions during *Trypanosoma cruzi* infection: trans-sialidase rides the show. Biochim. Biophys. Acta (BBA)-Molecular Basis Dis. 1866, 165692. doi: 10.1016/j.bbadis.2020.165692 PMC7819670 31972227

[B14] Castro-Borges W. Wilson R. A. (2022). Schistosome proteomics: updates and clinical implications. Expert Rev. Proteomics, 19, 1–15. doi: 10.1080/14789450.2022.2142565 36331139

[B15] Cavalcante T. Medeiros M. M. Mule S. N. Palmisano G. Stolf B. S. (2021). The role of sialic acids in the establishment of infections by pathogens, with special focus on *Leishmania* . Front. Cell. Infection Microbiol. 11, 671913. doi: 10.3389/fcimb.2021.671913 PMC8155805 34055669

[B16] Chaussabel D. Pascual V. Banchereau J. (2010). Assessing the human immune system through blood transcriptomics. BMC Biol. 8, 1–14. doi: 10.1186/1741-7007-8-84 20619006 PMC2895587

[B17] Chiang C.-P. Caulfield J. (1989a). The binding of human low-density lipoproteins to the surface of schistosomula of *Schistosoma mansoni* is inhibited by polyanions and reduces the binding of anti-schistosomal antibodies. Am. J. Pathol. 134, 1007–1018. 2719071 PMC1879897

[B18] Chiang C.-P. Caulfield J. (1989b). Human lipoprotein binding to schistosomula of *Schistosoma mansoni.* displacement by polyanions, parasite antigen masking, and persistence in young larvae. Am. J. Pathol. 135 1015–1024. 2596569 PMC1880489

[B19] Corfield T. (1992). Bacterial sialidases–roles in pathogenicity and nutrition. Glycobiology 2, 509–521. doi: 10.1093/glycob/2.6.509 1472757

[B20] Crocker P. R. Paulson J. C. Varki A. (2007). Siglecs and their roles in the immune system. Nat. Rev. Immunol. 7, 255–266. doi: 10.1038/nri2056 17380156

[B21] Da’dara A. A. Bhardwaj R. Ali Y. B. Skelly P. J. (2014). Schistosome tegumental ecto-apyrase (SmATPDase1) degrades exogenous pro-inflammatory and pro-thrombotic nucleotides. PeerJ 2, e316. doi: 10.7717/peerj.316 24711968 PMC3970803

[B22] Da’dara A. A. De Laforcade A. M. Skelly P. J. (2016). The impact of schistosomes and schistosomiasis on murine blood coagulation and fibrinolysis as determined by thromboelastography (TEG). J. Thromb. Thrombolysis 41, 671–677. doi: 10.1007/s11239-015-1298-z 26573180 PMC5467217

[B23] Da’dara A. A. Siddons G. Icaza M. Wang Q. Skelly P. J. (2017). How schistosomes alter the human serum proteome. Mol. Biochem. Parasitol. 215, 40–46. doi: 10.1016/j.molbiopara.2016.12.007 28011341 PMC5474353

[B24] Dagenais M. Gerlach J. Q. Collins Iii J. J. Atkinson L. E. Mousley A. Geary T. G.. (2021). Analysis of *Schistosoma mansoni* extracellular vesicles surface glycans reveals potential immune evasion mechanism and new insights on their origins of biogenesis. Pathogens 10, 1401. doi: 10.3390/pathogens10111401 34832557 PMC8617790

[B25] Dagenais M. Gerlach J. Q. Geary T. G. Long T. (2022). Sugar coating: Utilisation of host serum sialoglycoproteins by *Schistosoma mansoni* as a potential immune evasion mechanism. Pathogens 11, 426. doi: 10.3390/pathogens11040426 35456101 PMC9030049

[B26] Danckwardt S. Hentze M. W. Kulozik A. E. (2013). Pathologies at the nexus of blood coagulation and inflammation: thrombin in hemostasis, cancer, and beyond. J. Mol. Med. 91, 1257–1271. doi: 10.1007/s00109-013-1074-5 23955016 PMC3825489

[B27] de Bont C. M. Boelens W. C. Pruijn G. J. (2019). NETosis, complement, and coagulation: a triangular relationship. Cell. Mol. Immunol. 16, 19–27. doi: 10.1038/s41423-018-0024-0 29572545 PMC6318284

[B28] de la Torre-Escudero E. Bennett A. P. Clarke A. Brennan G. P. Robinson M. W. (2016). Extracellular vesicle biogenesis in helminths: more than one route to the surface? Trends Parasitol. 32, 921–929. doi: 10.1016/j.pt.2016.09.001 27720334

[B29] de la Torre-Escudero E. Gerlach J. Q. Bennett A. P. Cwiklinski K. Jewhurst H. L. Huson K. M.. (2019). Surface molecules of extracellular vesicles secreted by the helminth pathogen *Fasciola hepatica* direct their internalisation by host cells. PloS Negl. Trop. Dis. 13, e0007087. doi: 10.1371/journal.pntd.0007087 30657764 PMC6355031

[B30] Del Valle A. Jones B. F. Harrison L. M. Chadderdon R. C. Cappello M. (2003). Isolation and molecular cloning of a secreted hookworm platelet inhibitor from adult *Ancylostoma caninum* . Mol. Biochem. Parasitol. 129, 167–177. doi: 10.1016/S0166-6851(03)00121-X 12850261

[B31] De Marco Verissimo C. Jewhurst H. L. Dobó J. Gál P. Dalton J. P. Cwiklinski K. (2022). *Fasciola hepatica* is refractory to complement killing by preventing attachment of mannose binding lectin (MBL) and inhibiting MBL-associated serine proteases (MASPs) with serpins. PloS Pathog. 18, e1010226. doi: 10.1371/journal.ppat.1010226 35007288 PMC8782513

[B32] Diosdado A. Simón F. Morchón R. González-Miguel J. (2020). *Dirofilaria immitis* possesses molecules with anticoagulant properties in its excretory/secretory antigens. Parasitology 147, 559–565. doi: 10.1017/S0031182020000104 31992384 PMC10317642

[B33] Elzoheiry M. Da’dara A. A. Bhardwaj R. Wang Q. Azab M. S. El-Kholy E.-S. I.. (2018b). Intravascular *Schistosoma mansoni* cleave the host immune and hemostatic signaling molecule sphingosine-1-phosphate *via* tegumental alkaline phosphatase. Front. Immunol. 9, 1746. doi: 10.3389/fimmu.2018.01746 30105025 PMC6077193

[B34] Elzoheiry M. Da’dara A. A. Delaforcade A. M. El-Beshbishi S. N. Skelly P. J. (2018a). The essential ectoenzyme SmNPP5 from the human intravascular parasite *Schistosoma mansoni* is an ADPase and a potent inhibitor of platelet aggregation. Thromb. Haemostasis 118, 979–989. doi: 10.1055/s-0038-1641715 29669386 PMC12434806

[B35] Eneva R. Engibarov S. Abrashev R. Krumova E. Angelova M. (2021). Sialic acids, sialoconjugates and enzymes of their metabolism in fungi. Biotechnol. Biotechnol. Equip. 35, 364–375. doi: 10.1080/13102818.2021.1879678

[B36] Eyayu T. Zeleke A. J. Seyoum M. Worku L. (2020). Basic coagulation profiles and platelet count among *Schistosoma mansoni*-infected adults attending sanja primary hospital, Northwest Ethiopia. Res. Rep. Trop. Med. 11, 27. doi: 10.2147/RRTM.S244912 32368171 PMC7184861

[B37] Figueiredo B. C. Da’dara A. A. Oliveira S. C. Skelly P. J. (2015). Schistosomes enhance plasminogen activation: the role of tegumental enolase. PloS Pathog. 11, e1005335. doi: 10.1371/journal.ppat.1005335 26658895 PMC4676649

[B38] Gillis-Germitsch N. Kockmann T. Asmis L. M. Tritten L. Schnyder M. (2021). The *Angiostrongylus vasorum* excretory/secretory and surface proteome contains putative modulators of the host coagulation. Front. Cell. Infection Microbiol. 11, 1052. doi: 10.3389/fcimb.2021.753320 PMC8593241 34796127

[B39] Giorgi M. E. de Lederkremer R. M. (2011). Trans-sialidase and mucins of Trypanosoma cruzi: an important interplay for the parasite. Carbohydr. Res., 346, 1389–1393. doi: 10.1016/j.carres.2011.04.006 21645882

[B40] Glaus T. M. Sigrist N. Hofer-Inteeworn N. Kuemmerle-Fraune C. Mueller C. Geissweid K.. (2016). Unexplained bleeding as primary clinical complaint in dogs infected with *Angiostrongylus vasorum* . Schweizer Archiv für Tierheilkunde 158, 701–709. doi: 10.17236/sat00088 27707683

[B41] González-Miguel J. Morchón R. Mellado I. Carretón E. Montoya-Alonso J. A. Simón F. (2012). Excretory/secretory antigens from *Dirofilaria immitis* adult worms interact with the host fibrinolytic system involving the vascular endothelium. Mol. Biochem. Parasitol. 181, 134–140. doi: 10.1016/j.molbiopara.2011.10.010 22050927

[B42] Haapasalo K. Meri T. Jokiranta T. S. (2009). *Loa loa* microfilariae evade complement attack *in vivo* by acquiring regulatory proteins from host plasma. Infection Immun. 77, 3886–3893. doi: 10.1128/IAI.01583-08 PMC2738024 19528206

[B43] Hambrook J. R. Hanington P. C. (2021). Immune evasion strategies of schistosomes. Front. Immunol. 11, 624178. doi: 10.3389/fimmu.2020.624178 33613562 PMC7889519

[B44] Hirst N. L. Nebel J.-C. Lawton S. P. Walker A. J. (2020). Deep phosphoproteome analysis of *Schistosoma mansoni* leads development of a kinomic array that highlights sex-biased differences in adult worm protein phosphorylation. PloS Negl. Trop. Dis. 14, e0008115. doi: 10.1371/journal.pntd.0008115 32203512 PMC7089424

[B45] Hokke C. H. van Diepen A. (2017). Helminth glycomics–glycan repertoires and host-parasite interactions. Mol. Biochem. Parasitol. 215, 47–57. doi: 10.1016/j.molbiopara.2016.12.001 27939587

[B46] Horta M. Ramalho-Pinto F. Fatima M. (1991). Role of human decay-accelerating factor in the evasion of *Schistosoma mansoni* from the complement-mediated killing *in vitro* . J. Exp. Med. 174, 1399–1406. doi: 10.1084/jem.174.6.1399 1720809 PMC2119036

[B47] International Helminth Genomes Consortium (2019). Comparative genomics of the major parasitic worms. Nat. Genet. 51, 163–174. 30397333 10.1038/s41588-018-0262-1 PMC6349046

[B48] James S. L. Abate D. Abate K. H. Abay S. M. Abbafati C. Abbasi N.. (2018). Global, regional, and national incidence, prevalence, and years lived with disability for 354 diseases and injuries for 195 countries and territories 1990–2017: a systematic analysis for the global burden of disease study 2017. Lancet 392, 1789–1858. doi: 10.1016/S0140-6736(18)32279-7 30496104 PMC6227754

[B49] Joiner K. Sher A. Gaither T. Hammer C. (1986). Evasion of alternative complement pathway by *Trypanosoma cruzi* results from inefficient binding of factor b. Proc. Natl. Acad. Sci. 83, 6593–6597. doi: 10.1073/pnas.83.17.6593 2944112 PMC386550

[B50] Jolodar A. Fischer P. Bergmann S. Büttner D. W. Hammerschmidt S. Brattig N. W. (2003). Molecular cloning of an α-enolase from the human filarial parasite *Onchocerca volvulus* that binds human plasminogen. Biochim. Biophys. Acta (BBA)-Gene Structure Expression 1627, 111–120. doi: 10.1016/S0167-4781(03)00083-6 12818429

[B51] Kemp W. M. Merritt S. C. Bogucki M. S. Rosier J. G. Seed J. R. (1977). Evidence for adsorption of heterospecific host immunoglobulin on the tegument of *Schistosoma mansoni* . J. Immunol. 119, 1849–1854. doi: 10.4049/jimmunol.119.5.1849 915283

[B52] Khoo K.-H. Nieto A. Morris H. R. Dell A. (1997). Structural characterization of the n-glycans from *Echinococcus granulosus* hydatid cyst membrane and protoscoleces. Mol. Biochem. Parasitol. 86, 237–248. doi: 10.1016/S0166-6851(97)00036-4 9200129

[B53] Kleifeld O. Doucet A. Prudova A. Schilling O. Kainthan R. K. Starr A. E.. (2010). Isotopic labeling of terminal amines in complex samples identifies protein n-termini and protease cleavage products. Nat. Biotechnol. 28, 281–288. doi: 10.1038/nbt.1611 20208520

[B54] Knox D. (2007). Proteinase inhibitors and helminth parasite infection. Parasite Immunol. 29, 57–71. doi: 10.1111/j.1365-3024.2006.00913.x 17241394

[B55] Kuipers M. E. Nolte-’T Hoen E. N. van der Ham A. J. Ozir-Fazalalikhan A. Nguyen D. L. De Korne C. M.. (2020). DC-SIGN mediated internalisation of glycosylated extracellular vesicles from *Schistosoma mansoni* increases activation of monocyte-derived dendritic cells. J. Extracellular Vesicles 9, 1753420. doi: 10.1080/20013078.2020.1753420 32489529 PMC7241508

[B56] Kushner A. West W. P. Khan Suheb M. Z. Pillarisetty L. S. (2019). Virchow triad (Treasure Island (FL): StatPearls Publishing). 30969519

[B57] Lee J. J. Dissanayake S. Panico M. Morris H. R. Dell A. Haslam S. M. (2005). Mass spectrometric characterisation of *Taenia crassiceps* metacestode n-glycans. Mol. Biochem. Parasitol. 143, 245–249. doi: 10.1016/j.molbiopara.2005.06.002 16024105

[B58] Leontovyč A. Ulrychová L. O’donoghue A. J. Vondrášek J. Marešová L. Hubálek M.. (2018). SmSP2: A serine protease secreted by the blood fluke pathogen *Schistosoma mansoni* with anti-hemostatic properties. PloS Negl. Trop. Dis. 12, e0006446. doi: 10.1371/journal.pntd.0006446 29677188 PMC5931690

[B59] Loukas A. Jones M. K. King L. T. Brindley P. J. Mcmanus D. P. (2001). Receptor for Fc on the surfaces of schistosomes. Infection Immun. 69, 3646–3651. doi: 10.1128/IAI.69.6.3646-3651.2001 PMC98357 11349025

[B60] Maizels R. M. Bundy D. A. Selkirk M. E. Smith D. F. Anderson R. M. (1993). Immunological modulation and evasion by helminth parasites in human populations. Nature 365, 797–805. doi: 10.1038/365797a0 8413664

[B61] Maizels R. M. McSorley H. J. (2016). Regulation of the host immune system by helminth parasites. J. Allergy Clin. Immunol. 138, 666–675. doi: 10.1016/j.jaci.2016.07.007 27476889 PMC5010150

[B62] Maizels R. M. Smits H. H. McSorley H. J. (2018). Modulation of host immunity by helminths: the expanding repertoire of parasite effector molecules. Immunity 49, 801–818. doi: 10.1016/j.immuni.2018.10.016 30462997 PMC6269126

[B63] McSorley H. J. Hewitson J. P. Maizels R. M. (2013). Immunomodulation by helminth parasites: defining mechanisms and mediators. Int. J. Parasitol. 43, 301–310. doi: 10.1016/j.ijpara.2012.11.011 23291463

[B64] McVeigh P. Cwiklinski K. Garcia-Campos A. Mulcahy G. O’neill S. M. Maule A. G.. (2018). *In silico* analyses of protein glycosylating genes in the helminth *Fasciola hepatica* (liver fluke) predict protein-linked glycan simplicity and reveal temporally-dynamic expression profiles. Sci. Rep. 8, 1–15. doi: 10.1038/s41598-018-29673-3 30076319 PMC6076252

[B65] Mebius M. M. Van Genderen P. J. Urbanus R. T. Tielens A. G. De Groot P. G. Van Hellemond J. J. (2013). Interference with the host haemostatic system by schistosomes. PloS Pathog. 9, e1003781. doi: 10.1371/journal.ppat.1003781 24385897 PMC3873443

[B66] Medzhitov R. Janeway J. C.A. (2002). Decoding the patterns of self and nonself by the innate immune system. Science 296, 298–300. doi: 10.1126/science.1068883 11951031

[B67] Mehr K. Withers S. G. (2016). Mechanisms of the sialidase and trans-sialidase activities of bacterial sialyltransferases from glycosyltransferase family 80. Glycobiology 26, 353–359. doi: 10.1093/glycob/cwv105 26582604

[B68] Meningher T. Barsheshet Y. Ofir-Birin Y. Gold D. Brant B. Dekel E.. (2020). Schistosomal extracellular vesicle-enclosed miRNAs modulate host T helper cell differentiation. EMBO Rep. 21, e47882. doi: 10.15252/embr.201947882 31825165 PMC6944914

[B69] Meri T. Jokiranta T. S. Hellwage J. Bialonski A. Zipfel P. F. Meri S. (2002). *Onchocerca volvulus* microfilariae avoid complement attack by direct binding of factor h. J. Infect. Dis. 185, 1786–1793. doi: 10.1086/340649 12085326

[B70] Molehin A. J. Gobert G. N. Mcmanus D. P. (2012). Serine protease inhibitors of parasitic helminths. Parasitology 139, 681–695. doi: 10.1017/S0031182011002435 22310379

[B71] Moreno Y. Nabhan J. F. Solomon J. Mackenzie C. D. Geary T. G. (2010). Ivermectin disrupts the function of the excretory-secretory apparatus in microfilariae of *Brugia malayi* . Proc. Natl. Acad. Sci. 107, 20120–20125. doi: 10.1073/pnas.1011983107 21041637 PMC2993382

[B72] Newton W. L. (1968). Longevity of an experimental infection with *Dirofilaria immitis* in a dog. J. Parasitol. 54, 187–188. doi: 10.2307/3276912 5641045

[B73] Pappas P. (1988). The relative roles of the intestines and external surfaces in the nutrition of monogeneans, digeneans and nematodes. Parasitology 96, S105–S121. doi: 10.1017/S0031182000086005 3287286

[B74] Pearce E. Sher A. (1987). Mechanisms of immune evasion in schistosomiasis. Contributions to Microbiol. Immunol. 8, 219–232. 3304833

[B75] Pereira-Chioccola V. L. Acosta-Serrano A. Correia De Almeida I. Ferguson M. Souto-Padron T. Rodrigues M. M.. (2000). Mucin-like molecules form a negatively charged coat that protects *Trpanosoma cruzi* trypomastigotes from killing by human anti-alpha-galactosyl antibodies. J. Cell Sci. 113, 1299–1307. doi: 10.1242/jcs.113.7.1299 10704380

[B76] Previato J. Andrade A. F. Pessolani M. C. V. Mendonça-Previato L. (1985). Incorporation of sialic acid into *Trypanosoma cruzi* macromolecules. a proposal for a new metabolic route. Mol. Biochem. Parasitol. 16, 85–96. doi: 10.1016/0166-6851(85)90051-9 2412116

[B77] Quinzo M. Perteguer M. Brindley P. J. Loukas A. Sotillo J. (2022). Transgenesis in parasitic helminths: a brief history and prospects for the future. Parasites Vectors 15, 1–16. doi: 10.1186/s13071-022-05211-z 35346328 PMC8962113

[B78] Ravidà A. Aldridge A. M. Driessen N. N. Heus F. A. Hokke C. H. O’neill S. M. (2016). *Fasciola hepatica* surface coat glycoproteins contain mannosylated and phosphorylated n-glycans and exhibit immune modulatory properties independent of the mannose receptor. PloS Negl. Trop. Dis. 10, e0004601. doi: 10.1371/journal.pntd.0004601 27104959 PMC4841591

[B79] Rempel H. Calosing C. Sun B. Pulliam L. (2008). Sialoadhesin expressed on IFN-induced monocytes binds HIV-1 and enhances infectivity. PloS One 3, e1967. doi: 10.1371/journal.pone.0001967 18414664 PMC2288672

[B80] Royo F. Cossío U. De Angulo A. R. Llop J. Falcon-Perez J. M. (2019). Modification of the glycosylation of extracellular vesicles alters their biodistribution in mice. Nanoscale 11, 1531–1537. doi: 10.1039/C8NR03900C 30623961

[B81] Rubin-de-Celis S. S. Uemura H. Yoshida N. Schenkman S. (2006). Expression of trypomastigote trans-sialidase in metacyclic forms of *Trypanosoma cruzi* increases parasite escape from its parasitophorous vacuole. Cell. Microbiol. 8, 1888–1898. doi: 10.1111/j.1462-5822.2006.00755.x 16824037

[B82] Rumjanek F. D. Campos E. G. Carlos C. (1988). Evidence for the occurrence of LDL receptors in extracts of schistosomula of *Schistosoma mansoni* . Mol. Biochem. Parasitol. 28, 145–152. doi: 10.1016/0166-6851(88)90062-X 3367933

[B83] Santoro F. Ouaissi M. A. Pestel J. Capron A. (1980). Interaction between *Schistosoma mansoni* and the complement system: binding of C1q to schistosomula. J. Immunol. 124, 2886–2891. doi: 10.4049/jimmunol.124.6.2886 6989909

[B84] Santos R. Ursu O. Gaulton A. Bento A. P. Donadi R. S. Bologa C. G.. (2017). A comprehensive map of molecular drug targets. Nat. Rev. Drug Discovery 16, 19–34. doi: 10.1038/nrd.2016.230 27910877 PMC6314433

[B85] Schauer R. (1985). Sialic acids and their role as biological masks. Trends Biochem. Sci. 10, 357–360. doi: 10.1016/0968-0004(85)90112-4

[B86] Schauer R. Kamerling J. P. (2011). The chemistry and biology of trypanosomal trans-sialidases: virulence factors in chagas disease and sleeping sickness. ChemBioChem 12, 2246–2264. doi: 10.1002/cbic.201100421 21956798

[B87] Schauer R. Kamerling J. P. (2018). Exploration of the sialic acid world. Adv. Carbohydr. Chem. Biochem. 75, 1–213. doi: 10.1016/bs.accb.2018.09.001 30509400 PMC7112061

[B88] Schenkman S. Jiang M.-S. Hart G. W. Nussenzweig V. (1991). A novel cell surface trans-sialidase of *Trypanosoma cruzi* generates a stage-specific epitope required for invasion of mammalian cells. Cell 65, 1117–1125. doi: 10.1016/0092-8674(91)90008-M 1712251

[B89] Severi E. Hood D. W. Thomas G. H. (2007). Sialic acid utilization by bacterial pathogens. Microbiology 153, 2817–2822. doi: 10.1099/mic.0.2007/009480-0 17768226

[B90] Shakhnovich E. A. King S. J. Weiser J. N. (2002). Neuraminidase expressed by *Streptococcus pneumoniae* desialylates the lipopolysaccharide of *Neisseria meningitidis* and *Haemophilus influenzae*: a paradigm for interbacterial competition among pathogens of the human respiratory tract. Infection Immun. 70, 7161–7164. doi: 10.1128/IAI.70.12.7161-7164.2002 PMC133026 12438402

[B91] Shao S. Sun X. Chen Y. Zhan B. Zhu X. (2019). Complement evasion: An effective strategy that parasites utilize to survive in the host. Front. Microbiol. 10, 532. doi: 10.3389/fmicb.2019.00532 30949145 PMC6435963

[B92] Skelly P. J. Da’dara A. A. Li X.-H. Castro-Borges W. Wilson R. A. (2014). Schistosome feeding and regurgitation. PloS Pathog. 10, e1004246. doi: 10.1371/journal.ppat.1004246 25121497 PMC4133383

[B93] Skelly P. J. Nation C. S. Da’dara A. A. (2022). *Schistosoma mansoni* and the purinergic halo. Trends Parasitol. 38, 1080–1088. doi: 10.1016/j.pt.2022.09.001 PMC9669209 36182536

[B94] Skelly P. Tielens A. Shoemaker C. (1998). Glucose transport and metabolism in mammalian-stage schistosomes. Parasitol. Today 14, 402–406. doi: 10.1016/S0169-4758(98)01319-2 17040830

[B95] Skelly P. J. Wilson R. A. (2006). Making sense of the schistosome surface. Adv. Parasitol. 63, 185–284. doi: 10.1016/S0065-308X(06)63003-0 17134654

[B96] Smithers S. Terry R. Hockley D. (1969). Host antigens in schistosomiasis. proceedings of the royal society of London. Ser. B. Biol. Sci. 171, 483–494. doi: 10.1098/rspb.1969.0007 4388253

[B97] Sohanpal B. K. El-Labany S. Lahooti M. Plumbridge J. A. Blomfield I. C. (2004). Integrated regulatory responses of fimB to n-acetylneuraminic (sialic) acid and GlcNAc in *Escherichia coli* K-12. Proc. Natl. Acad. Sci. 101, 16322–16327. doi: 10.1073/pnas.0405821101 15534208 PMC526197

[B98] Sohanpal B. K. Friar S. Roobol J. Plumbridge J. A. Blomfield I. C. (2007). Multiple co-regulatory elements and IHF are necessary for the control of fimB expression in response to sialic acid and n-acetylglucosamine in *Escherichia coli* K-12. Mol. Microbiol. 63, 1223–1236. doi: 10.1111/j.1365-2958.2006.05583.x 17238917

[B99] Sotillo J. Toledo R. Mulvenna J. Loukas A. (2017). Exploiting helminth–host interactomes through big data. Trends Parasitol. 33, 875–888. doi: 10.1016/j.pt.2017.06.011 28734897

[B100] Souri M. Osaki T. Ichinose A. (2015). The non-catalytic b subunit of coagulation factor XIII accelerates fibrin cross-linking. J. Biol. Chem. 290, 12027–12039. doi: 10.1074/jbc.M114.608570 25809477 PMC4424339

[B101] Staniunas R. J. Hammerberg B. (1982). Diethylcarbamazine-enhanced activation of complement by intact microfilariae of *Dirofilaria immitis* and their *in vitro* products. J. Parasitol. 68, 809–816. doi: 10.2307/3280987 7131186

[B102] Steenbeek S. C. Pham T. V. De Ligt J. Zomer A. Knol J. C. Piersma S. R.. (2018). Cancer cells copy migratory behavior and exchange signaling networks *via* extracellular vesicles. EMBO J. 37, e98357. doi: 10.15252/embj.201798357 29907695 PMC6068466

[B103] Tarleton R. Kemp W. (1981). Demonstration of IgG-fc and C3 receptors on adult *Schistosoma mansoni* . J. Immunol. 126, 379–384. doi: 10.4049/jimmunol.126.1.379 7451978

[B104] Torpier G. Capron A. Ouaissi M. (1979). Receptor for IgG (Fc) and human β2-microglobulin on *S. mansoni* schistosomula. Nature 278, 447–449. doi: 10.1038/278447a0 88014

[B105] Tritten L. Ballesteros C. Beech R. Geary T. G. Moreno Y. (2021a). Mining nematode protein secretomes to explain lifestyle and host specificity. PloS Negl. Trop. Dis. 15, e0009828. doi: 10.1371/journal.pntd.0009828 34587193 PMC8504978

[B106] Tritten L. Gillis-Germitsch N. Kockmann T. Schnyder M. (2021b). Quantitative proteomics analysis of *Angiostrongylus vasorum*-induced alterations in dog serum sheds light on the pathogenesis of canine angiostrongylosis. Sci. Rep. 11, 1–11. doi: 10.1038/s41598-020-79459-9 33431914 PMC7801463

[B107] Tsang V. Hubbard W. J. Damian R. T. (1977). Coagulation factor XIIa (activated hageman factor) inhibitor from adult *Schistosoma mansoni* . Am. J. Trop. Med. Hygiene 26, 243–247. doi: 10.4269/ajtmh.1977.26.243 848647

[B108] Ubersax J. A. Ferrell J. J.E. (2007). Mechanisms of specificity in protein phosphorylation. Nat. Rev. Mol. Cell Biol. 8, 530–541. doi: 10.1038/nrm2203 17585314

[B109] Vedamurthy G. Sahoo S. Devi I. Murugavel S. Joshi P. (2015). The n-terminal segment of glyceraldehyde-3-phosphate dehydrogenase of *Haemonchus contortus* interacts with complements C1q and C3. Parasite Immunol. 37, 568–578. doi: 10.1111/pim.12273 26332726

[B110] Vogl G. Lesiak I. Jensen D. Perkhofer S. Eck R. Speth C.. (2008). Immune evasion by acquisition of complement inhibitors: the mould *Aspergillus* binds both factor h and C4b binding protein. Mol. Immunol. 45, 1485–1493. doi: 10.1016/j.molimm.2007.08.011 17915330 PMC5654503

[B111] Wang Q. Da’dara A. A. Skelly P. J. (2017). The human blood parasite *Schistosoma mansoni* expresses extracellular tegumental calpains that cleave the blood clotting protein fibronectin. Sci. Rep. 7, 1–13. doi: 10.1038/s41598-017-13141-5 29018227 PMC5635006

[B112] Wang Q. Da’dara A. A. Skelly P. J. (2018). The blood fluke *Schistosoma mansoni* cleaves the coagulation protein high molecular weight kininogen (HK) but does not generate the vasodilator bradykinin. Parasites Vectors 11, 1–10. doi: 10.1186/s13071-018-2704-0 29540224 PMC5853081

[B113] Warren L. (1963). The distribution of sialic acids in nature. Comp. Biochem. Physiol. 10, 153–171. doi: 10.1016/0010-406X(63)90238-X 14109742

[B114] Wiedemann M. Voehringer D. (2020). Immunomodulation and immune escape strategies of gastrointestinal helminths and schistosomes. Front. Immunol. 11, 572865. doi: 10.3389/fimmu.2020.572865 33042153 PMC7527441

[B115] Williams C. Pazos R. Royo F. González E. Roura-Ferrer M. Martinez A.. (2019). Assessing the role of surface glycans of extracellular vesicles on cellular uptake. Sci. Rep. 9, 1–14. doi: 10.1038/s41598-019-48499-1 31417177 PMC6695415

[B116] Williams C. Royo F. Aizpurua-Olaizola O. Pazos R. Boons G.-J. Reichardt N.-C.. (2018). Glycosylation of extracellular vesicles: current knowledge, tools and clinical perspectives. J. Extracellular Vesicles 7, 1442985. doi: 10.1080/20013078.2018.1442985 29535851 PMC5844028

[B117] Wu K. Zhai X. Huang S. Jiang L. Yu Z. Huang J. (2021). Protein kinases: potential drug targets against *Schistosoma japonicum* . Front. Cell. Infection Microbiol. 11. doi: 10.3389/fcimb.2021.691757 PMC8282181 34277472

[B118] Yang J. Qiu C. Xia Y. Yao L. Fu Z. Yuan C.. (2010). Molecular cloning and functional characterization of *Schistosoma japonicum* enolase which is highly expressed at the schistosomulum stage. Parasitol. Res. 107, 667–677. doi: 10.1007/s00436-010-1913-z 20512506

[B119] Yong W. Das P. (1983). Acquisition of host proteins by the tegument of *Schistosoma mansoni* recovered from rats. Z. für Parasitenkunde 69, 53–60. doi: 10.1007/BF00934010 6340359

[B120] Zakeri A. Hansen E. P. Andersen S. D. Williams A. R. Nejsum P. (2018). Immunomodulation by helminths: intracellular pathways and extracellular vesicles. Front. Immunol. 9, 2349. doi: 10.3389/fimmu.2018.02349 30369927 PMC6194161

